# Preleukemia and Leukemia-Initiating Cell Activity in inv(16) Acute Myeloid Leukemia

**DOI:** 10.3389/fonc.2018.00129

**Published:** 2018-04-26

**Authors:** John Anto Pulikkan, Lucio Hernán Castilla

**Affiliations:** Department of Molecular, Cell and Cancer Biology, University of Massachusetts Medical School, Worcester, MA, United States

**Keywords:** myeloid, leukemia, CBFB-MYH11, CBFβ-SMMHC, preleukemia, clonal evolution, leukemia-initiating cell, stem cells

## Abstract

Acute myeloid leukemia (AML) is a collection of hematologic malignancies with specific driver mutations that direct the pathology of the disease. The understanding of the origin and function of these mutations at early stages of transformation is critical to understand the etiology of the disease and for the design of effective therapies. The chromosome inversion inv(16) is thought to arise as a founding mutation in a hematopoietic stem cell (HSC) to produce preleukemic HSCs (preL-HSCs) with myeloid bias and differentiation block, and predisposed to AML. Studies in mice and human AML cells have established that inv(16) AML follows a clonal evolution model, in which preL-HSCs expressing the fusion protein CBFβ–SMMHC persist asymptomatic in the bone marrow. The emerging leukemia-initiating cells (LICs) are composed by the inv(16) and a heterogeneous set of mutations. In this review, we will discuss the current understanding of inv(16) preleukemia development, and the function of CBFβ–SMMHC related to preleukemia progression and LIC activity. We also discuss important open mechanistic questions in the etiology of inv(16) AML.

## Introduction

The core-binding factor (CBF) transcription factor has critical roles in hematopoietic stem cell (HSC) maintenance and differentiation by regulating expression of genes associated with cell fate decisions and proliferation in lymphoid and myeloid compartments (1). The CBF has two core subunits and is frequently associated with cofactors that modulate their activity or provide target specificity. The subunit CBFβ increases RUNX affinity to DNA approximately 40-fold and stabilizes RUNX protein from proteasome degradation (2–4). The subunit RUNX (encoded by either *RUNX1, RUNX2*, and *RUNX3* genes) binds to DNA at promoters and enhancers (consensus sequence TGYGGT). RUNX is the docking subunit that interacts with CBFβ and cofactors and has the nuclear localization signal (5, 6).

From the clinical and mechanistic points of view, AML is a collection of hematologic malignancies marked by specific driver mutations. *RUNX1* and *CBFB* genes are recurrently mutated in AML. Although a variety of mutations in *RUNX1* have been described in hematologic malignancies, the only rearrangement associated with *CBFB* is the pericentric inversion inv(16)(p13q22), henceforth inv(16), in leukemia (7–9). The inv(16) generates the fusion gene *CBFB-MYH11*, encoding the leukemia fusion protein CBFβ–SMMHC (10). Most of inv(16) AML cases have a myelomonocytic morphology with abnormal eosinophils and are classified as AML subtype M4-Eo, and in rare occasions as AML subtypes M0, M1, M2, and M5 [French–American–British system (11)]. In spite of the morphology, the inv(16) AML transcriptome clusters as a single entity, suggesting a common underlying molecular alteration (12). The World Health Organization grouped “inv(16) AML” within the “AML with recurrent genetic abnormalities” based on genetic, molecular, and clinical features (13).

The name preleukemia has been used in different contexts in hematologic malignancies and has evolved in the past years (14). The preleukemic HSCs (preL-HSCs) can be considered as HSCs with inv(16) as a founding mutation that generate a clonal expansion of myeloid progenitor cells primed for leukemia (15). In this review, we summarize the current understanding in preleukemia progression of inv(16) AML.

## CBFβ–SMMHC Domains That Regulate Leukemia Development

**Figure 1:**
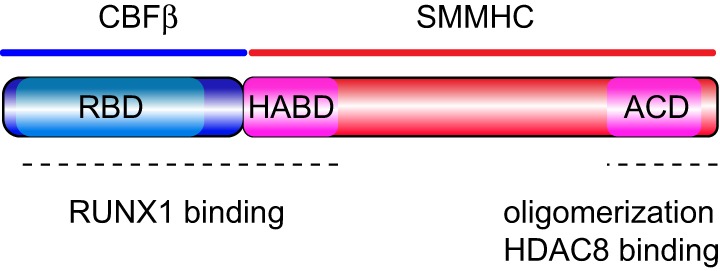
Protein organization of CBFβ–SMMHC. Schematic representation of the CBFβ–SMMHC fusion protein, including the RUNX1-binding domain (RBD) at the N-terminus of CBFβ, the *high-affinity binding domain* (HABD) at the proximal end of SMMHC, and the *assembly competence domain* (ACD) near the C-terminus in the SMMHC region. Functional regions are marked with dash line at the bottom.

Two domains in CBFβ–SMMHC that are critical for its leukemogenic function: the *RUNX binding domain* (RBD) and the *assembly competence domain* (ACD) (Figure 1). The RBD, corresponding to the 135 N-terminal amino acids of CBFβ region at the N-terminus of the fusion protein, binds to the RUNX factors (16, 17). Genetic evidence, using *Cbfb^+/MYH11^* knock-in mice, revealed that RUNX activity is essential for CBFβ–SMMHC-associated leukemia function. Accordingly, reduction of *Runx1* or *Runx2* expression inhibited CBFβ–SMMHC-mediated differentiation block in embryos and leukemia onset in mice (18, 19). Furthermore, the increase in Runx2 levels reduced leukemia median latency (20). RUNX1 also interacts with the *high-affinity binding domain* (HABD), at the N-terminus of SMMHC. Surprisingly, RUNX1 binds to CBFβ–SMMHC with approximately 10-fold higher affinity to than to CBFβ. Its dual interaction with the RBD and HABD provides a rationale for the observed dominant negative function of the fusion protein outcompeting CBFβ for RUNX1 binding (21). A later study using *Cbfb^+/MYH11d179-221^* knock-in mice expressing CBFβ–SMMHC lacking the HABD established that HABD regulates myeloid differentiation induced by CBFβ–SMMHC but it may actually inhibit leukemia by altering the LIC pool (22). These findings have direct clinical significance because although the majority of inv(16) AML cases include HABD sequence in the *CBFB-MYH11* transcripts, fraction of cases lack HABD sequence due to a different breakpoint on th*e MYH11* part of inv(16). The 28 amino acid ACD near the C-terminus is responsible for the oligomerization of CBFβ–SMMHC molecules and formation of filament structures (23–25). The ACD activity is needed for CBFβ–SMMHC’s ability to inhibit myeloid differentiation, regulate the expression of CBF targets, and to reduce cell cycle and its nuclear localization *in vitro* (26, 27). Two recent studies using different inv(16) leukemia models have established that the ACD is essential for the expansion of preleukemic cells and for leukemia development (28, 29). Furthermore, the analysis of preleukemic progenitor cells revealed that ACD activity is critical for block in early B-cell differentiation but that sequences outside the ACD in the fusion protein impair T-cell differentiation. Finally, the C-terminal 95 amino acid region of CBFβ–SMMHC, which includes the ACD, binds to the histone deacetylase HDAC8 (30, 31). This interaction is essential for the inv(16) LIC activity because HDAC8 deacetylates p53, rendering it inactive, and modulates the transcription repression function of the fusion protein (31). Finally, inhibition of CBFβ–SMMHC binding to these factors may efficiently reduce preL-HSC and LIC activities, resulting in promising candidates for targeted therapies (32).

## The Origin of inv(16) Preleukemia

Our understanding on the origin of AML is still evolving, and in general terms it seems to follow a clonal evolution model (33–35). In inv(16) AML, a small number of studies have tested the origin of inv(16) preL-HSCs in the hematopoietic system. Studies using a *breakpoint backtracking* approach evaluated whether the inv(16) breakpoint identified in the DNA of a patient’s inv(16) AML sample is present in the patient’s neonatal bloodspot (also called Guthrie card or neonatal heel prick). Two studies identified the inv(16) breakpoint in the bloodspots, demonstrating that preL-HSCs can originate during fetal development and persist quiescent for years (4 to 10 in these studies) before AML diagnosis (36, 37). In a third case with inv(16) AML, the bloodspot analysis was negative suggesting that either the preL-HSCs were infrequent (below the sensitivity of the assay) or that inv(16) occurred postnatally. Of note, since backtracking studies have only been done in pediatric inv(16) AML cases, it is unknown if inv(16) preL-HSCs are prenatal in adult AML. Breakpoint backtracking studies for other leukemia fusion genes, such as RUNX1-RUNX1T1 and TEL-RUNX1, have also confirmed the prenatal origin of preL-HSCs (38–40).

The screening of leukemia fusion transcripts using RT-PCR analysis in healthy individuals revealed that 1 of 10 cord blood and 1 of 58 peripheral blood samples from adult individuals were *CBFB-MYH11* positive (41). These results lack statistical value due to the reduced sample size but suggest that preL-HSCs may persist in the hematopoietic system for years. However, the use of RT-PCR has been disputed because of the challenge in identifying the chromosome breakpoints in fusion transcript positive samples of healthy individuals (42, 43), result that could be explained by transplicing (44, 45).

## The inv(16) Preleukemic Progression

The identification of inv(16) preL-HSCs and progenitor cells has important therapeutic value because it is considered the source of leukemia development, drug resistance, and relapse. From a conceptual point, it would shed light on the etiology of disease progression. Studies in mice where allelic *CBFB-MYH11* expression is activated in hematopoietic cells have established that leukemia is preceded by a preleukemic period of 4 to 6 months, and the median leukemia latency can be delayed or render incomplete penetrance by reducing the number of HSCs expressing *CBFB-MYH11* (28, 46). Furthermore, chimeric mice (composed by *Cbfb^MYH11/+^* embryonic stem cells and wild-type blastocyst cells) expressing *CBFB-MYH11* in a fraction of their HSCs remained healthy and only developed AML when treated with chemical or retroviral mutagenesis (20, 47). These studies determined that *CBFB-MYH11* expression is necessary but not sufficient for leukemogenesis.

**Figure 2:**
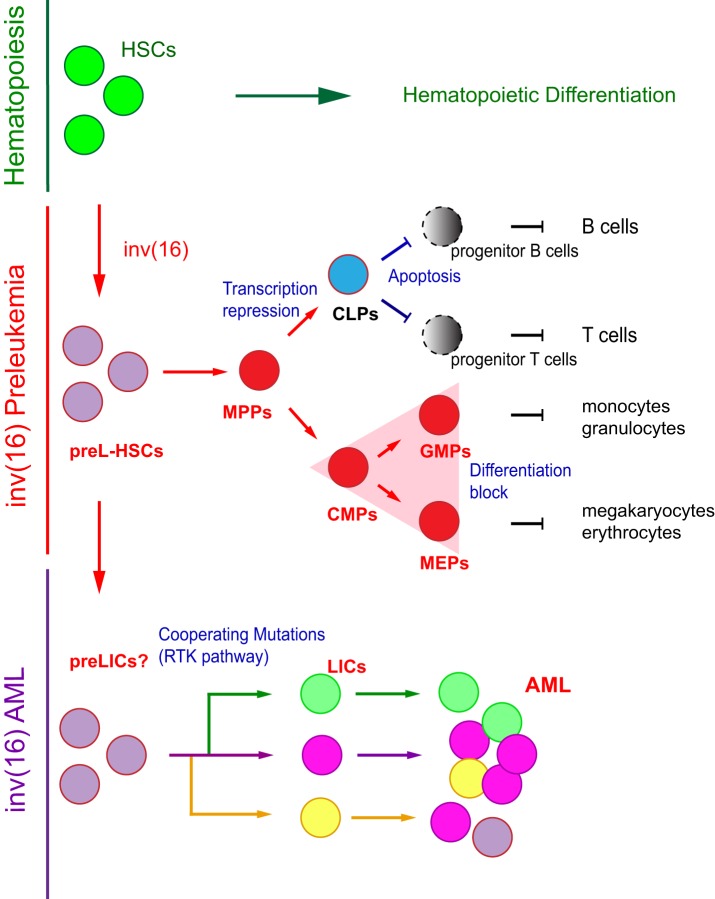
Model of inv(16) associated preleukemia. Normal hematopoiesis is summarized on top. Preleukemia hematopoietic stem cell (preL-HSCs) with inv(16) and myeloid preleukemic progenitors (triangle compartment) are shown in red; preL-HSC-derived lymphoid progenitors and lineages with differentiation block are depicted in gray. Clonal expansion of leukemic cells from a putative preleukemia initiating cell with inv(16) and “cooperating” mutations are shown in green, violet, and orange.

During the preleukemic period, *Cbfb^MYH11/+^* HSCs produce abnormal hematopoiesis, with cell compartment-specific defects, myeloid bias, and multilineage differentiation block (Figure 2). In the early progenitor compartment, CBFβ–SMMHC expression induces expansion of the short-term HSCs and multipotential progenitor (MPP) cells, although the frequency of long-term HSCs (putative preLICs) is unchanged, indicating that CBFβ–SMMHC may modulate factors associated with cell-fate decisions (46, 48).

These HSCs undergo normal early lymphoid differentiation, with normal numbers of common lymphoid progenitors (CLPs) but with reduced expression of transcription factors (*Ebf, E2a*, and *Pax5*) responsible for the commitment to B and T cell differentiation (49). During B cell commitment, CBFβ–SMMHC induces a marked reduction in pre-pro B cells and in pre-B cells due to apoptosis. These blocks are probably due to repression of RUNX1 activity because similar deficiencies were reported in *Cbfb*- and *Runx1*-knockout mice (50–52). Similarly, differentiation of CBFβ–SMMHC-expressing CLPs to T cell progenitors showed reduced cell number and viability of the double-negative compartments (53). Its repressive function in the production of lymphoid cells in humans was confirmed by fluorescent *in situ* hybridization analysis of lineage sorted inv(16) AML cells (54). Interestingly, the inability of inv(16) preL-HSCs to differentiate to B and T cells provides a mechanism for the myeloid leukemia bias observed in inv(16) AML.

CBFβ–SMMHC-expressing preL-HSCs undergo partial myeloid differentiation, displaying a mixed myeloid-erythroid progenitors (MEPs) and common myeloid progenitors (CMPs) immunophenotype [Figure 2, red triangle (46)] and a predominant blast/myeloblast and promyelocyte morphology. Contrary to its strong apoptotic activity on the lymphoid compartment, CBFβ–SMMHC increases the viability of preleukemic myeloid cells and enhances their resistance to genotoxic stress (46, 48, 55). The mechanism by which CBFβ–SMMHC blocks myeloid differentiation is not fully understood. Expression studies suggest that levels of a number of myeloid factors are affected by the fusion protein, including the repression of transcription factors that regulate myeloid lineage commitment (e.g., Cebpa, PU.1, Sox4, Hoxa9, and Irf8), some of which are known Runx1 targets. On the other hand, upregulated factors in preleukemic myeloid cells are implicated in survival and proliferation pathways [e.g., Csf2rb, il1rl1, Fosb, c-Jun, Erg1, and WT1 (28, 55, 56)]. Despite significant progress in this area, it is not clear which of these targets directs differentiation block in inv(16) AML. For example, the myeloid transcription factors C/EBPα and PU.1, both CBF targets, act as tumor suppressors in AML (57–59). In addition, Sox4 has been shown to function as an oncogene in *Cebpa*-mutated AML (60). On the other hand, expression of the colony stimulating factor 2 receptor beta (Csf2rb), is expressed in myeloid progenitor cells of *Cbfb^56M/+^;Mx1Cre* mice and has a negative correlation with preL-HSC activity (56).

Transplantation studies of inv(16) preleukemic myeloid cells in mice, revealed that preleukemic cells could not induce leukemia in irradiated recipients (28, 46), indicating that preL-HSCs are not LICs, and that “cooperating” mutations are needed for leukemia transformation. Alternatively, the LIC activity is possibly present at a frequency below 1 in 20,000 preleukemic cells. Therefore, as rare preL-HSCs differentiate to myelomonocytic preleukemic cells and accumulate in the MEP/GMP compartment, additional events seem to be required for leukemia transformation.

## LIC Activity in inv(16) AML

Our understanding of LIC activity is evolving rapidly with the application of new technologies. Using targeted sequencing techniques in diagnostic inv(16) AML samples, studies have identified an average of 3 (range = 0–6) secondary mutations per sample (61, 62). The majority of inv(16) AML “cooperating” mutations are in genes encoding components of the RTK pathway, with predominance *KIT, FLT3*, and *NRAS* (63–65). In contrast, mutations in genes associated with components of cohesin or chromatin complexes are rare (62, 66). Evidence for inv(16) and PU.1 associated leukemia in mice suggests that transformation of preleukemic progenitors could be enhanced by mutations that “weaken” its oncogenic repression activity, thereby moving the differentiation block to a more mature myeloid progenitor that is permissive for transformation (22, 67). This model has been previously illustrated using mouse models for *CEBPA*-mutated AML. *Cebpa*-null mice show differentiation block at the CMPs and remain leukemia free. However, in mice carrying a leukemia-associated *Cebpa* point mutation, differentiation continues to stall at the committed myeloid progenitors and mice succumb with myeloid leukemia (68, 69). The molecular mechanism underlying this perplexing function, however, remains unknown.

inv(16) AML follows the clonal evolution model, whereby *de novo* inv(16) AML samples at diagnosis are composed of multiple leukemia subclones, which have emerged from the same preL-HSCs (Figure 2). The subclones share the founding mutation but have a different combination of “cooperating” mutations (70). Each subclone originates from an independent LIC with a different mutation combination and sensitivity to therapies. In addition to the leukemia subclones, the *de novo* AML sample includes preL-HSCs with reduced chemosensitivity, and that may serve as precursors for the expansion of resistant clones at relapse (15, 71). Longitudinal (diagnosis/relapse-matched) studies of AML mutational landscape using whole-genome sequencing have confirmed the clonal evolution model in inv(16) AML (72, 73). In these studies, the AML samples contained 1 to 18 “cooperating” mutations (mean = 6), corresponding to 1 to 3 mutations per subclone. In addition, inv(16) was found in all subclones at both stages of disease progression while a heterogeneity in the “cooperating” mutations indicated clonal evolution and differential sensitivity to therapy. Studies in mice have validated the basic premise of this model in inv(16) AML (48, 74, 75), and the weak LIC activity reported in human and mouse studies was validated in titration dilution transplantation experiments (48).

## inv(16) as a “Cooperating” Mutation in Leukemia

The inv(16) is predominantly a founding mutation that predisposes to *de novo* AML. Accumulating case reports have identified inv(16) in other hematologic malignancies clearly showing that this inversion, at a low frequency, can also originate as a “cooperating” mutation in the progression of other cancers. The inv(16) can emerge in BCR-ABL-positive chronic myelogenous leukemia (CML) cases transitioning to blast crisis (76–80). The appearance of a inv(16)-positive predominant clone is accompanied by a switch to an immature monocytic morphology and dysplastic eosinophils. In CML cells, the occurrence of inv(16) predicts rapid evolution and poor outcome (77, 80). In addition, inv(16) has been reported in 1–2% of tAML cases that progressed from MDS or solid tumors (81). Probably due to the paucity of these cases, the mechanism of CBFβ–SMMHC function in the LICs from CML-PB or tAML cases has not been studied. However, the understanding of its function when acting as a “cooperating” mutation could open new insights on leukemia progression. It should be noted that CBFβ–SMMHC function in the LIC of CML-chronic phase (i.e., with active proliferative signals) or post-therapy HSCs/MDS (i.e., with higher mutation content) may involve different targets.

## Conclusion and Perspectives

The inv(16) is a somatic mutation that activates CBFβ–SMMHC expression in an HSC, either *in utero* or after birth. Indirect evidence suggests that these preL-HSCs can perdure for years to produce a clonal population with myeloid bias and impaired differentiation. Over time, the preL-HSCs are primed for leukemogenesis after acquiring a relatively small number of “cooperating” mutations, predominantly in components of the RTK pathway. The finding that mutations in genes associated with epigenetic complexes, frequently mutated in other AMLs, are practically absent in inv(16) AML suggests that CBFβ–SMMHC function may deregulate chromatin dynamics.

Future studies are endowed to demonstrate whether preL-HSCs can produce preleukemia initiating cells in inv(16) AML (Figure 2). The application of new technologies, such as single cell analysis, next-generation sequencing, CRISPR/Cas9 editing in primary hematopoietic stem and progenitor cells, pharmacology, and sophisticated animal models will greatly enhance our understanding of inv(16) preleukemia biology and minimal residual disease. Considering that each LIC in diagnostic inv(16) AML has a small number of mutations and a heterogeneity of mutations between diagnosis and relapse cases, targeted therapies inhibiting CBFβ–SMMHC binding to RUNX1 and HDAC8, and combination with RTK inhibitors may result in effective treatment. Pharmacologic approaches directly inhibiting specific signals could be valuable to define which components drive preleukemia to leukemia progression. In addition, little is known on the preL-HSC activity in relation with the microenvironment and how changes in the immune system affect LIC activity. The role of RUNX1 in inv(16) AML seems perplexing, as reduction in Runx1 levels decreases leukemia development in mice but loss of RUNX1 levels induce cell death in inv(16) AML cells. It is, therefore, possible that reduction in RUNX1 levels may be required for preleukemia formation and transition to LICs. New strategies designed to force increase in RUNX1 expression may help define new RUNX targets with potential antileukemia functions. The dependence of the RBD and ACD domains in CBFβ–SMMHC in preleukemia and LIC activity clearly indicate that SMMHC-multimerization and RUNX1 binding are critical leukemogenic functions. Interestingly, mutations in both domains interfere with the nuclear localization of the fusion protein. Hence, the development of strategies to directly interfere with the nuclear import of CBFβ–SMMHC may abrogate its leukemic activity. Finally, the study of the inv(16) LIC activity in *de novo* AML versus tAML and CML-blast crisis may shed mechanistic insights on the function of the fusion protein in cells with different mutation composition and proliferation capacity.

## Author Contributions

All authors listed have contributed to the preparation and editing of the work and approved it for publication.

## Conflict of Interest Statement

The authors declare that the research was conducted in the absence of any commercial or financial relationships that could be construed as a potential conflict of interest.
